# Red cell aspartate aminotransferase saturation with oral pyridoxine intake

**DOI:** 10.1590/S1516-31802005000200004

**Published:** 2005-03-02

**Authors:** Marilena Oshiro, Kimiyo Nonoyama, Raimundo Antônio Gomes Oliveira, Orlando Cesar de Oliveira Barretto

**Keywords:** Pyridoxine, Pyridoxal phosphate, Aspartate aminotransferase, Sideroblastic anemia, Myelofibrosis, Aspartato aminotransferase, Anemia sideroblástica, Mielofibrose, Piridoxina, Fosfato de piridoxal

## Abstract

**CONTEXT AND OBJECTIVE:**

The coenzyme of aspartate aminotransferase is pyridoxal phosphate, generated from fresh vegetables containing pyridoxine. Vitamin B_6_-responsive sideroblastic anemia, myelofibrosis and Peyronie's syndrome respond to high pyridoxine doses. The objective was to investigate the oral pyridoxine oral dose that would lead to maximized pyridoxal phosphate saturation of red cell aspartate aminotransferase.

**DESIGN AND SETTING:**

Controlled trial, in Hematology Division of Instituto Adolfo Lutz.

**METHODS:**

Red cell aspartate aminotransferase activity was assayed (before and after) in normal volunteers who were given oral pyridoxine for 15-18 days (30 mg, 100 mg and 200 mg daily). *In vitro* study of blood from seven normal volunteers was also performed, with before and after assaying of aspartate aminotransferase activity.

**RESULTS:**

The *in vivo* study showed increasing aspartate aminotransferase saturation with increasing pyridoxine doses. 83% saturation was reached with 30 mg daily, 88% with 100 mg, and 93% with 200 mg after 20 days of oral supplementation. The *in vitro* study did not reach 100% saturation.

**CONCLUSIONS:**

Neither *in vivo* nor *in vitro* study demonstrated thorough aspartate aminotransferase saturation with its coenzyme pyridoxal phosphate in red cells, from increasing pyridoxine supplementation. However, the 200-mg dose could be employed safely in vitamin B_6-_responsive sideroblastic anemia, myelofibrosis and Peyronie's syndrome treatment. Although maximum saturation in circulating red cells is not achieved, erythroblasts and other nucleated and cytoplasmic organelles containing cells certainly will reach thorough saturation, which possibly explains the results obtained in these diseases.

## RESUMO

**CONTEXTO E OBJETIVO:**

A enzima aspartato aminotransferase apresenta o piridoxal fosfato como coenzima, oriunda da piridoxina existente em alimentos vegetais frescos. A anemia sideroblástica responsiva à vitamina B_6_, mielofibrose e síndrome de Peyronie respondem a altas doses de piridoxina. O objetivo foi investigar a máxima resposta da aspartato aminotransferase à suplementação oral com piridoxina.

**TIPO DE ESTUDO E LOCAL:**

Experimento controlado, na Seção de Hematologia, Instituto Adolfo Lutz.

**MÉTODOS:**

A atividade da aspartato aminotransferase eritrocitária foi determinada (antes e após) em voluntários que receberam suplementação por 15-18 dias (30 mg, 100 mg e 200 mg diariamente). Estudo *in vitro* também foi realizado, com sangue de sete indivíduos. As atividades enzimáticas antes e após a incubação foram determinadas, seguindo o mesmo protocolo do estudo *in vivo.*

**RESULTADOS:**

O estudo *in vivo* revelou um aumento gradativo da saturação da aspartato aminotransferase com doses crescentes de piridoxina. 83% de saturação foi alcançada com 30 mg diariamente, 88% com 100 mg e 93% com 200 mg. O estudo *in vitro* não revelou saturação de 100%.

**CONCLUSÕES:**

Tanto *in vivo* quanto *in vitro*, não se revelou saturação completa da aspartato aminotransferase por sua coenzima piridoxal-5-fosfato nos eritrócitos. Entretanto, a dose de 200 mg diariamente poderia ser empregada com segurança no tratamento da anemia sideroblástica, mielofibrose e síndrome de Peyronie. Embora a saturação máxima nos eritrócitos não seja atingida, os eritroblastos e outras células nucleadas que contenham as organelas citoplasmáticas certamente atingirão a saturação completa, possivelmente à razão dos resultados obtidos nas doenças citadas.

## INTRODUCTION

The action of aspartate aminotransferase, also known as glutamic-oxaloacetic transaminase (GOT), is dependent on its coenzyme pyridoxal-5-phosphate, which is a pyridoxine metabolite. Pyridoxine, or vitamin B_6_, is the natural precursor found in fresh vegetables, and its conversion to pyridoxal-5-phosphate is carried out mainly in the liver but also in the red cells.^1–3^ Aspartate aminotransferase activity is usually assayed by means of spectrophotometry, and such assaying reflects the apoenzyme activity afforded by the pyridoxal-5-phosphate available in red cells. When pyridoxal-5-phosphate is added to the reagent system, it occupies all the sites that exist for this enzyme and the activity reaches a maximum value, thereby revealing the aspartate aminotransferase holoenzyme activity. Thus, in the case of dietary pyridoxine deficiency there is a large increase in activity when pyridoxal-5-phosphate is added to the reagent system. Therefore, the aspartate aminotransferase activity depends on the quality of the food intake, and this fact has been widely used to ascertain the vitamin B_6_ nutritional status. The poorer the diet is, the more activation is observed. The hematological diseases vitamin B_6_-responsive sideroblastic anemia and myelofibrosis, and also the urological disease Peyronie's syndrome respond to high pyridoxine doses.^4,5^

An activity coefficient has been proposed, based on the ratio between the aspartate aminotransferase activity before pyridoxal-5-phosphate addition and the activity after its addition to the reagent system. Thus, if the activity coefficient were high, it would mean a large vitamin B_6_ dietary deficiency.^4,6–9^

The normal values for the activity coefficient range from ≥ 1.5 to ≤ 2.0,^4–8^ depending on the countries where these surveys have been performed. This diversity in activity coefficients observed among different countries is thought to reflect different levels of aspartate aminotransferase saturation with vitamin B_6_ that is dependent upon dietary habits, with foods that may or may not be rich in pyridoxine. However, when vitamin B_6_ supplementation takes place, there is increased aspartate aminotransferase activity and a concomitant reduction in the activity coefficient.^10^

The aspartate aminotransferase saturation with its coenzyme that is observed among individuals receiving their usual food does not reach 100% (activity coefficient = 1). Theoretically, aspartate aminotransferase that was very active would facilitate and improve the crucial transamination of aspartate and alpha-ketoglutarate to glutamic acid and oxaloacetate, which feed the aerobic oxidative mitochondrial pathway that is present in erythroblasts and all other tissues. This leads to the question of whether total saturation of aspartate aminotransferase would be reached if oral vitamin supplementation were given to normal individuals. The present study was designed to address this question through the administration of increasing oral doses of pyridoxine to normal individuals.

## MATERIAL AND METHODS

### *In vivo* study

The subjects were normal adult male and female volunteers working at Instituto Adolfo Lutz, São Paulo. Their ages ranged from 24 to 54 years and they were not taking any vitamin supplements. All these volunteers gave their informed consent for their participation. They were divided into three groups:

- Group A: 15 volunteers who were given 30 mg of pyridoxine daily for 20 days;
- Group B: 15 volunteers who were given 100 mg of pyridoxine daily for 20 days;
- Group C: 18 volunteers who were given 200 mg of pyridoxine daily for 20 days.

The period of 20 days was chosen as the minimum period of time for allowing all the red cells to utilize the pyridoxine, from young to senescent circulating red cells.

### *In vitro* study

To evaluate the input of pyridoxine to the red cells and its metabolism, 20 ml of blood were collected from seven normal volunteers and divided between sterile tubes. 0.3 mM pyridoxine was added to every tube except for one control tube. Before incubation, the aspartate aminotransferase activity was assayed. Following this, all the tubes were incubated at 37° C, with constant stirring for 24 hours, and aspartate aminotransferase assaying was performed again, twice. For the sample preparation and enzyme assaying, 5 ml of blood were drawn and collected in anticoagulant solution of acid-citrate-dextrose. The samples were washed three times in saline at 4° C, and lysed in stabilizing solution (0.7 μM β-mercaptoethanol, 2 μM nicotinamide adenine dinucleotide phosphate and 0.27 M ethylenediamine tetraacetic acid). The red cell aspartate aminotransferase activity, with and without pyridoxal phosphate, was assayed using a Gilford 2451 spectrophotometer at 37° C, according to the method described by Beutler^11^ (1984). The percentage saturation of aspartate aminotransferase with its coenzyme pyridoxal-5-phosphate was obtained by means of the ratio between aspartate aminotransferase activity without pyridoxal-5-phosphate and aspartate aminotransferase activity after pyridoxal-5-phosphate was added to the reagent system, multiplied by 100. The non-parametric Mann-Whitney statistical method was utilized, with a significance level of 5%.

## RESULTS

The *in vivo* data obtained from the groups A, B and C are shown in Tables 1, 2 and 3 respectively. Table 1 shows that group A, which was given 30 mg daily for 20 days, underwent an enzyme activity change from 76% to a maximum saturation of 83%. Table 2 shows that group B, which was given 100 mg daily for 20 days, had an increase from 71% to 88% saturation. Table 3 shows that group C, which received 200 mg daily for 20 days, had an increase from 83% to a maximum observed value of 93%.

**Table 1:** Aspartate aminotransferase activity (UI/gHb/min at 37° C) in the blood of normal volunteers with and without the addition of pyridoxal-5-phosphate to the reagent system, before and after daily intake of 30 mg of pyridoxine for 20 days

Pyridoxine supplementation	Without pyridoxal-5-phosphate in the reagent system	With pyridoxal-5-phosphate in the reagent system	% saturation
Before	3.6 ± 0.54	4.7 ± 0.64	76 ± 5.51
After	4.7 ± 0.68	5.6 ± 0.67	83 ± 6.04
p-value	< 0.0001	0.0001	< 0.0001

**Table 2:** Aspartate aminotransferase activity (UI/gHb/min at 37° C) in the blood of normal volunteers with and without the addition of pyridoxal-5-phosphate to the reagent system, before and after daily intake of 100 mg of pyridoxine for 20 days

Pyridoxine supplementation	Without pyridoxal-5-phosphate in the reagent system	With pyridoxal-5-phosphate in the reagent system	% saturation
Before	3.83 ± 0.79	5.45 ± 1.16	71 ± 10.42
After	5.75 ± 0.89	6.56 ± 1.10	88 ± 6.98
p-value	< 0.0001	0.0012	< 0.0001

**Table 3:** Aspartate aminotransferase activity (UI/gHb/min at 37° C) in the blood of normal volunteers with and without the addition of pyridoxal-5-phosphate to the reagent system, before and after daily intake of 200 mg of pyridoxine for 20 days

Pyridoxine supplementation	Without pyridoxal-5-phosphate in the reagent system	With pyridoxal-5-phosphate in the reagent system	% saturation
After	5.7 ± 1.52	6.1 ± 1.45	93 ± 4.96.
p-value	< 0.0001	< 0.0001	< 0.0001

The data obtained from the *in vitro* experiment are displayed in Table 4 and show that, after 24 hours of incubation, the red cells with pyridoxine presented an increase in saturation from 77% to 93%.

**Table 4:** *In vitro* aspartate aminotransferase activity (IU/gHb/min at 37° C) in the blood of normal volunteers with and without the addition of pyridoxal-5-phosphate to the reagent system, before and after whole blood incubation with 0.3 mM pyridoxine for 24 hours (n = 7)

Incubation time	Without pyridoxine addition to the whole blood (control)			With pyridoxine addition to the whole blood		
	Without PLP in the reagent system	With PLP in the reagent system	% saturation	Without PLP in the reagent system	With PLP in the reagent system	% saturation
0 hours	4.3 ± 0.70	5.7 ± 0.65	76 ± 6.15	4.1 ± 0.76	5.3 ± 0.79	77 ± 5.39
24 hours	3.7 ± 0.57	4.5 ± 0.59	81 ± 6.24	5.8 ± 0.55	6.3 ± 0.69	93 ± 4.87
p-value	0.0005	0.0005	0.0522	0.0159	0.0313	0.0165

*PLP = pyridoxal-5-phosphate.*

## DISCUSSION

The *in vivo* study (Tables 1, 2 and 3) showed that there were significant aspartate aminotransferase activity increases in all the individuals who received pyridoxine supplementation. It can be seen that, when no pyridoxal-5-phosphate was added to the reagent system, an increase in activity was observed in relation to the samples from individuals before treatment with pyridoxine. This activity increase might be ascribable to the activation of the existing aspar-tate aminotransferase apoenzymes, induced by the greater availability of the coenzyme inside the red cells, which could stabilize the enzyme, or by a decrease in enzyme hydrolysis.^11^ It is known that the pyridoxal-5-phosphate synthesized in the red cells is linked to hemoglobin,^5^ which also protects it from hydrolysis.

However, it can be seen from the aspartate aminotransferase activity with pyridoxal-5-phosphate added to the reagent system that there was a significant increase in enzyme activity, in comparison with the activity at the beginning of the treatment. This suggests that there was an increase in aspartate amino-transferase molecule synthesis per cell (or per mg of protein) when these individuals’ diets were supplemented with oral pyridoxine. In addition to this effect, the pyridoxine in excess might induce greater synthesis of pyridoxine kinase, which is an enzyme involved in pyridoxine metabolism.^10^

Tables 1, 2 and 3 show that the increasing pyridoxine doses led to higher aspartate aminotransferase saturation with its coenzyme. The greatest saturation found was 93%, obtained with 200 mg daily for 20 days. This result was similar to results reported by Solomon and Hillman^10^ (1978) for 200 mg daily for 14 weeks of treatment. It is reasonable to suggest that there is a maximum physiological saturation around this value.

In the present study, the maximum supplementation dose was 200 mg daily, since higher doses may lead to neuropathy.^3^ However, higher doses have been utilized in the treatment of vitamin B_6_-responsive sideroblastic anemia, myelofibrosis and Peyronie's syndrome.^4,5^ Nevertheless, the reason for these therapeutic effects remains unknown, except that pyridoxine works as a coenzyme with ALA-synthetase for delta-aminolevulinic acid synthesis, which is a heme precursor of hemoglobin, cytochromes, catalase, triptophan oxygenase, etc. In vitamin B_6_-responsive sideroblastic anemia, doses of 300 mg to 600 mg have been recommended for controlling the disease.^12^ We would suggest that a lower dose of around 200 mg would be sufficient to achieve the same therapeutic effect, since red cells are considered to be a good mirror of what is happening in other tissues. These considerations may be extended to myelofibrosis and Peyronie's syndrome as well. The reason for seeking to minimize the dose needed for achieving maximum response to the treatment is that high doses may induce a maximized metabolic effect from all the enzymes and proteins that have heme as a prosthetic group. Such doses certainly act upon cells that have nucleated and cytoplasmic organelles, such as mitochondria, in which part of the heme biosynthesis occurs.

The maximum saturation of 93% for aspartate aminotransferase with its coenzyme pyridoxal-5-phosphate may be explained by the inability of the senescent red cells that comprise about 10% of all circulating red cells to carry out the pyridoxine to pyridoxal-5-phosphate conversion.

Generally speaking, pyridoxal phosphate has an extensive role in cells, such as in the metabolism of proteins, amino acids, fatty acids and glycogen,^13–15^ activation of steroid hormones,^14^ and synthesis of serotonin, taurine, dopamine, histamine and delta aminolevulinic acid.^7,16^ All cells could therefore benefit from high pyridoxine doses, provided that these do not exceed 200 mg daily, since this may be toxic.^3^

Table 4 (*in vitro* study) shows that there was increased aspartate aminotransferase activity in the red cells incubated with pyridoxine, and also increased aspartate aminotransferase saturation with its coenzyme pyridoxal-5-phosphate. It was discussed earlier that pyridoxine could increase the quantities of aspartate aminotransferase apoenzyme molecules, as well as other enzymes involved in pyridoxine metabolism. However, the mature red cells do not have all the cytoplasmic organelles and lack the ability to synthesize mRNA and proteins, except for reticulocytes, which still have functioning organelles for approximately 72 hours.^17^ In effect, it is known that during these 72 hours the reticulocytes synthesize about 20% of all hemoglobin that will be present in red cells. Moreover, what is observed in relation to hemoglobin may be extended to all other proteins and enzymes.

Another explanation for the activity increase *in vitro* could be the suggestion from Rose et al.^18^ (1973). In this, there would be activation of the pre-existing aspartate amino-transferase apoenzyme molecules, induced by the greater quantity of pyridoxal-5-phosphate synthesized through the greater supply of pyridoxine. There would also be stabilization of the enzyme, afforded by the higher pyridoxal-5-phosphate concentration. When the red cells are incubated with pyridoxine, the pyridoxine would enter the cells and would supply their metabolic needs.

Interestingly, the samples incubated without pyridoxine showed a significant decrease in aspartate aminotransferase activity after 24 hours. It can be suggested that the coenzyme presented changes, possibly by hydrolysis, as reported by Fonda^19^ (1992). As there is no pyridoxine in the extra-cellular environment, the pyridoxine transformation to the coenzyme pyridoxal-5-phosphate does not occur, and the aspartate aminotransferase activity decreases. This observation emphasizes how important the presence of pyridoxal-5-phosphate for aspartate aminotransferase metabolic function is, and hints that pyridoxal-5-phosphate is constantly degraded and that its continuous replacement is required for aspartate aminotransferase to be kept active. Another explanation would be the modifications to aspartate aminotransferase structure caused by pyridoxal-5-phosphate scarcity.^20^

It is known that aspartate aminotransferase activity decreases along with red cell aging in the blood circulation, which has been used to assess the concentration of young erythrocytes in the blood. In spite of the decrease in aspartate aminotransferase activity during the *in vitro* incubation, it was observed in this study that pyridoxine entered the red cells, and this may have caused the metabolic change of pyridoxine to pyridoxal-5-phosphate. 93% saturation was achieved after 24 hours of incubation with 0.39 mM pyridoxine at 37° C. Solomon^21^ (1982) reported that when red cells are incubated with pyridoxal-5-phosphate and pyridoxamine monophosphate, 100% saturation was obtained. This hinted that senescent red cells were not able to synthesize the coenzyme pyridoxal-5-phosphate, thus inhibiting thorough *in vivo* saturation with the coenzyme.

## CONCLUSION

The complete *in vivo* saturation of aspar-tate aminotransferase that is hypothetically possible with oral pyridoxine intake did not occur, even with high doses.

Neither the *in vivo* nor the *in vitro* study demonstrated thorough aspartate aminotransferase saturation with its coenzyme pyridoxal-5-phosphate by means of high doses of pyridoxine supplementation in circulating red cells. It can be suggested that there is a physiological limit to the saturation of aspartate aminotransferase in circulating red cells by means of oral pyridoxine intake, which we found to be 93%. Even the *in vitro* experiment demonstrated that complete apoenzyme saturation with its coenzyme was not achieved in circulating red cells. Nonetheless, a dose of 200 mg may be utilized safely in the treatment of vitamin B_6_-responsive sideroblastic anemia, myelofibrosis and Peyronie's syndrome. Although the maximum saturation in circulating red cells is not achieved, erythroblasts and other nucleated and cytoplasmic organelles containing cells certainly will reach thorough saturation, which is possibly the reason for the good results obtained in the cited diseases.
